# Joint Modulation of Facial Expression Processing by Contextual Congruency and Task Demands

**DOI:** 10.3390/brainsci9050116

**Published:** 2019-05-17

**Authors:** Luis Aguado, Karisa B. Parkington, Teresa Dieguez-Risco, José A. Hinojosa, Roxane J. Itier

**Affiliations:** 1 Facultad de Psicología, Universidad Complutense, 28223 Madrid, Spain; hinojosa@pluri.ucm.es; 2 Department of Psychology, University of Waterloo, Waterloo, ON N2L 3G1, Canada; kparkington@uwaterloo.ca (K.B.P.); ritier@uwaterloo.ca (R.J.I.); 3 Facultad de Psicología, Universidad Europea de Madrid, 28670 Madrid, Spain; teresa.dieguez@universidadeuropea.es; 4 Facultad de Lenguas y Educación, Universidad de Nebrija, 28015 Madrid, Spain

**Keywords:** facial expressions, situational context, ERPs, N170, EPN, LPP

## Abstract

Faces showing expressions of happiness or anger were presented together with sentences that described happiness-inducing or anger-inducing situations. Two main variables were manipulated: (i) congruency between contexts and expressions (congruent/incongruent) and (ii) the task assigned to the participant, discriminating the emotion shown by the target face (emotion task) or judging whether the expression shown by the face was congruent or not with the context (congruency task). Behavioral and electrophysiological results (event-related potentials (ERP)) showed that processing facial expressions was jointly influenced by congruency and task demands. ERP results revealed task effects at frontal sites, with larger positive amplitudes between 250–450 ms in the congruency task, reflecting the higher cognitive effort required by this task. Effects of congruency appeared at latencies and locations corresponding to the early posterior negativity (EPN) and late positive potential (LPP) components that have previously been found to be sensitive to emotion and affective congruency. The magnitude and spatial distribution of the congruency effects varied depending on the task and the target expression. These results are discussed in terms of the modulatory role of context on facial expression processing and the different mechanisms underlying the processing of expressions of positive and negative emotions.

## 1. Introduction

Facial expressions of emotion are highly relevant stimuli in human social interactions. Thus, it is not surprising that the cognitive and neural underpinnings of their processing have been the object of many studies. In our daily life, we rarely perceive facial expressions of our conspecifics isolated from any other stimuli. Instead, facial expressions are typically perceived in the context of specific social interactions that include many other relevant clues about the event itself and the person expressing the emotion. However, in most experimental studies facial expressions have been presented alone in the absence of any contextual information. In consequence, there is still limited evidence regarding the way in which contextual information modulates the processing of facial expressions of emotion.

In an attempt to reproduce the situated, contextual nature of social interactions, some recent studies have explored the impact of different types of contexts on the processing of facial expressions of emotion. In these studies, facial expression targets are presented in the context of intra-subject (e.g., prosody or body posture) or situational information (see [1,2] for reviews). In the present paper, we concentrate on the possible modulatory role of situational contexts on facial expression processing, where situational context refers to the information provided by the life event or social encounter in which the expressive behavior takes place. For example, the smile of a friend who has just told us about a personal misfortune will be processed and interpreted in a different way than that same smile in a casual conversation. 

Studies on contextual modulation have used contextual cues stimuli that can be affectively congruent or incongruent with the emotion expressed by a target face. Those stimuli include pictures with positive or negative valence [3,4,5], or sentences describing situations inducing specific emotions such as anger or joy [6,7,8]. The results of these studies have provided evidence of context influences on the processing of emotional faces that are apparent at both the behavioral and neural activity levels. For example, behavioral studies have reported slower and/or less accurate responses on trials in which the target and the context are emotionally incongruent [4,9]. Moreover, several studies using the event-related potential (ERP) technique have revealed modulations of neural activity that are dependent on the congruency between expression targets and picture or sentence contexts. These modulations have been observed at different post-stimulus onset times, beginning at early stages of perceptual face processing. This is the case for the face-sensitive N170 ERP component, a negative deflection that peaks around 170 ms over parieto-occipital sites that is considered the earliest reliable electrophysiological index of face encoding ([10,11,12], see [13] for a review). Amplitude modulations of the N170 by the congruency between contexts and expression targets have been observed in several studies [3,5,14]. These context effects, together with the sensitivity of the N170 to emotional expression (see [15] for a meta-analysis and review), suggest that relatively complex, context-dependent affective processing of face stimuli occurs already at early perceptual stages. 

Modulations driven by contextual congruency have also been shown on a later, post-perceptual ERP component—LPP (late positive potential). The LPP is a centro-parietal, sustained positive deflection that typically appears between 300 and 700 ms after stimulus onset and that shows increased amplitude in the presence of emotionally arousing relative to neutral stimuli (e.g., [16,17]). LPP is thought to reflect facilitated processing and encoding of relevant emotional stimuli, and thus it is not surprising that it has also been shown to be modulated by affective congruency. Enhanced positive amplitudes of LPP have been observed when a target face shows an emotional expression that is incongruent with the context in which it is presented [6,7] and in trials in which the prime and target stimuli are affectively incongruent in the affective priming paradigm [18,19,20]. 

A further, relatively early ERP component that is also sensitive to emotion is the EPN (early posterior negativity), a negativity detected over temporo-occipital sites that reaches maximal values around 300 ms after stimulus onset. This component has been found to respond to the emotional intensity of emotional pictures and words and is thought to reflect emotional facilitation of sensory processing (e.g., [21,22,23]). Moreover, in studies with facial expressions of emotion, enhanced amplitudes of the EPN are usually seen in response to angry and fearful faces compared to neutral and happy faces (e.g., [24,25,26]). In a broader sense, both the LPP and the EPN components have been considered to reflect relatively late, post-perceptual processes, aimed at enhancing continued encoding and processing motivationally relevant stimuli. As stated above, sensitivity of LPP to affective congruency has been shown in some studies. However, whether the EPN is also sensitive to affective congruency is still unknown. 

Priming and context studies with facial expressions of emotion have usually assigned the participants tasks that do not require explicit attention to the context or prime stimuli, such as emotion categorization or evaluation in terms of valence and arousal. Congruency effects observed under these conditions suggest that the affective valence of contexts and prime stimuli is automatically activated and influences processing of the target expression by means of implicit mechanisms. However, there is evidence that task demands can modify the way in which affective primes and contexts influence processing of target stimuli. This has been shown in the affective priming paradigm in which responses to affective words or pictures are influenced by their affective congruency with an immediately preceding prime (see [27] for a review). Several studies have shown that the affective priming effect, with slower and/or less accurate responses to the target on affectively incongruent trials, is observed in an evaluative task in which the target has to be categorized in terms of its valence but is significantly reduced or completely abolished when the task requires categorizing the target on the basis of non-affective features (e.g., [28,29,30]). Also relevant are the results of a study [31] that showed that attention to neutral picture contexts was promoted by the instruction to identify the emotion shown by a target face, compared to the instruction to make a general affective evaluation. More specifically, the participants showed better memory of the contexts after the emotion identification task. A direct demonstration of the effects of task demands also came from a recent behavioral study [9]. This study showed that the influence of affective sentence contexts on the response to target expressions varied depending on the specific task assigned to the participant. While an evaluative task (whether the face expression is positive or negative) produced only a weak affective congruency effect, an emotion categorization task (being able to discriminate between angry, fearful, and happy expressions) and a congruency categorization task (is the face expression congruent or incongruent with the context) both led to emotional congruency effects in which responses were slower and/or less accurate on (incongruent) trials in which the context and the target represented different specific emotions (e.g., fear and anger). These effects were especially strong in the congruency categorization task, in which the participants had to categorize the target expression as congruent or incongruent with the situation described by the sentence context. 

The evidence mentioned above suggests that different task instructions can selectively increase the saliency of different aspects of contextual information, thus modulating its influence on target processing. A main distinction would be between tasks that orient the participant to general affective properties of the target (evaluative tasks) and those that require attention to more specific affective properties (e.g., emotion categorization). An unsettled issue is the processing stage at which task demands exert their influence. While an effect of task demands at a given stage indicates that the underlying cognitive operation is subject to the influence of top-down processes, the absence of such an effect is more compatible with the operation of stimulus-driven processes. 

Due to its high temporal resolution, the ERP technique is ideally suited to track precisely the dynamics of brain activity underlying different cognitive tasks. In the study of Diéguez-Risco et al. [6], no congruency effects between contexts and target faces were found on the N170 component during an emotion categorization task. However, in a similar study in which the task required one to explicitly pay attention to the congruency between the context and the facial expression, the N170 was modulated by congruency [7], with larger negative amplitudes to faces showing expressions that were contextually incongruent. Although no firm conclusions can be drawn based on between-experiment comparisons, this discrepancy suggests that top-down processes driven by task demands might play a relevant role in the processing of affective congruency and that this influence could take place at an early perceptual processing stage such as that indexed by the N170. 

Using a within-subject design, the current study aimed at directly exploring the influence of the context on the processing of facial expressions of emotion under different task demands. EEG was recorded throughout the two tasks, and reaction times and accuracy were monitored. In each trial, participants were presented a context, consisting of a sentence describing an affectively positive or negative situation, displayed directly below a neutral face. The same individual face immediately followed this, expressing an emotion (joy or anger) as if reacting to the situation. In the emotion discrimination task, participants were simply asked to identify which emotion was expressed (joy or anger). In the congruency task, participants decided whether the emotion expressed by the face was congruent or incongruent with the situation described by the sentence. An important methodological aspect of this study was the monitoring of eye movements by the participants during their reading of each sentence, using an eye tracker. This ensured that, although the context was irrelevant for the emotion discrimination task, it was nonetheless read and thus, presumably, attended to. This aspect is important, given none of the prior studies in this field ever ensured that contextual sentences were read.

The comparison between the congruency and the emotion discrimination task allowed us to study the influence of top-down processes on emotional processing. Although in both task conditions efforts were made to ensure that the participants read the context sentence, explicit consideration of the relation between the context and the target face was required only in the congruency task. Thus, it may be assumed that contextual modulation of target processing would be driven by top-down processes only in the congruency task. As this task requires explicit consideration of the relation between the context and the target expression, congruency should have a maximal effect. Under these conditions, explicit and more effortful processing of the target expression in relation to its context should be revealed in stronger modulation of ERP components that are sensitive to the affective properties of stimuli and that have also been found to be modulated by affective congruency in previous studies. Based on this rationale, the amplitude of three ERP components—the N170, the EPN, and the LPP—was our main dependent variable.

## 2. Materials and Methods

### 2.1. Participants

Fifty-eight (58) undergraduate and graduate students from the University of Waterloo participated in this study for course credit or cash payment ($10/hour). All participants were fluent English speakers who reported normal or corrected-to-normal vision, and had lived in Canada and/or the USA for at least ten years. Participants did not have a history of psychological or neurological disorders, head trauma with loss of consciousness for more than five minutes, or epilepsy or seizures, and were not taking anti-psychotic medications or medications containing cortisone at the time of testing. This study was approved by a Human Research Ethics Board at the University of Waterloo (ORE #20113) and, in accordance with the Declaration of Helsinki, all participants provided informed written consent prior to starting the experiment. 

Data for 22 participants were rejected due to eye-tracking difficulties (could not calibrate the eye tracker, 10), completion of only one of the two experimental tasks (2), equipment malfunction (1), too few trials per condition due to extensive artifacts in the EEG recording (5), too few trials per condition due to eye movements during emotional face presentation (1), or too few trials due to errors or no responses (3). This resulted in a final sample of 36 participants (mean age = 21.83 years, SD = 4.02; 15 men, 21 women; and 31 right-handed). 

### 2.2. Stimuli

Greyscaled photographs of 10 models (five males, five females; models #: 02F, 07F, 08F, 09F, 10F, 21M, 23M, 25M, 26M, and 29M) from the NimStim face database [32] were used as target stimuli. Each model expressed both open- and closed-mouth expressions of anger and happiness, as well as a neutral expression, resulting in a total of 30 face images. Faces were cropped into ovals and placed on a grey background. These stimuli were the same as those used in Diéguez-Risco et al. [6]. Four additional face identities (each with neutral, happy, and angry expressions) were selected to be used in the practice phase. Images did not differ significantly in Root Mean Square (RMS) contrast (Mean RMS = 0.42, Standard Deviation SD = 0.008) or mean Pixel Intensity (PI) (Mean PI = 0.5, SD = 0.0004). 

Twenty short sentences describing emotion-inducing daily situations were used as contextual sentences. Half of these sentences described joy-inducing situations (positive sentences), and the other half described anger-inducing situations (negative sentences). The sentences used in the current ERP study were selected from a larger set, based on the data of a pilot behavioural study. In this pilot study, 51 participants (Mean age = 19.9 years, SD age = 3.3; 8 men, 43 women) categorized 30 sentences as joy-inducing or anger-inducing. Participants were required to indicate how intense (positive or negative) this emotion would be felt by the person going through the described situation (intensity rating) and how exciting or stressful this emotion would be (arousal rating). The final set of selected sentences (Appendix A) were categorized as happy-inducing or angry-inducing by at least 70% of these participants. The final positive and negative sentences were not significantly different in intensity (*t*(18) = 0.11, *p* = 0.91, mean difference = 0.042) or arousal (*t*(18) = −0.67, *p* = 0.51, and mean difference = −0.291) ratings. Mean intensity (on a scale from 1—very low to 9—very high) was 6.91 (Standard Error of the Mean SEM = 0.31) for positive sentences and 6.87 (SEM = 0.21) for negative sentences. Mean emotional arousal (on a scale from 1—very low to 9—very high) was 6.57 (SEM = 0.34) and 6.86 (SEM = 0.25) for negative and positive sentences, respectively. Eight additional sentences were used for the practice phase. 

### 2.3. Procedure

Participants sat in a sound- and electrically-attenuated Faraday cage, and were seated 70 cm from the display computer. All participants completed two different experimental tasks: an emotional categorization and a congruency categorization task, the order of which was counter-balanced across participants. 

**Figure 1:**
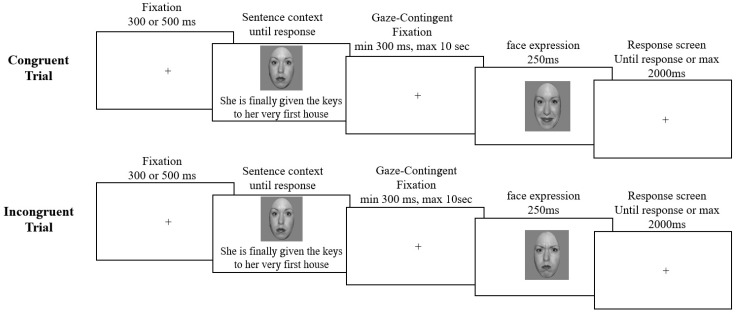
Exemplars of congruent and incongruent trials with angry and happy expressions (trials progression from left to right). In both tasks, a fixation cross was presented for 300 ms or 500 ms (jittered by 0–100 ms), followed by the presentation of a face with a neutral expression and a contextual sentence. After reading the sentence, participants pressed the spacebar, and a gaze-contingent fixation cross appeared in the centre of the screen. Participants were required to fixate on this cross for 300 ms, triggering the presentation of an emotional (angry or happy) face for 250 ms. If participants did not fixate on the cross within 10 seconds, the trial was aborted and a drift correction was performed. Following emotional face presentation, a response screen with a central fixation cross was presented until participants made a response, or for a maximum of 2000 ms. For the emotion task, participants indicated if the face was happy or angry. For the congruency task, participants indicated if the sentence was emotionally congruent or incongruent with the emotional face. Note: the sentence was written on one line only—displayed here on two lines for display purposes; furthermore, the NimStim model displayed here was not used in the study but is one for which we have publishing agreement.

In both tasks, a fixation cross was presented first, followed by a neutral face presented in the center of the screen and a contextual sentence (either positive or negative) situated underneath (Figure 1). Participants were told that, in each trial, the person shown had just gone through the situation described in the sentence, and that they should read each sentence carefully. The neutral face and contextual sentence remained on the screen until the participant pressed the spacebar to continue. This triggered the presentation of a central fixation cross, and participants were instructed to remain fixated on this cross in order to trigger the emotional face presentation. Once participants fixated on the cross for 300 ms, an emotional face (of the same individual), centered on the nasion, was presented for 250 ms. If participants did not fixate on the cross after ten seconds, the trial was aborted and a drift correction was performed before continuing to the next trial. Participants were instructed to stay focused on the nasion of the face (i.e., to not make eye movements during the emotional face presentation). A fixation cross was then presented in the centre of the screen for 2000 ms or until the participant made a behavioural response (whichever criterion was met first). In the emotion task, participants indicated whether the target face was happy or angry. In the congruency task, they judged whether the facial expression and sentence were congruent (i.e., whether the person expressed an emotion that would normally be appropriate given the situation described) or incongruent (i.e., the person expressed an emotion that would not normally be appropriate given the situation). Responses were recorded using a standard keyboard, and participants pressed the left and right arrow keys (counterbalanced across participants) with their right hand.

Each task consisted of 320 trials divided into four blocks. In each block, there were four main conditions: positive sentence–happy expression (happy congruent), negative sentence–angry expression (angry congruent), negative sentence–happy expression (happy incongruent), and positive sentence–angry expression (angry incongruent). Within each task, there were 80 trials across blocks for each of the four conditions. Within each block, each model’s facial expression was displayed four times (two congruent trials, and two incongruent trials), and there was a total of 20 trials for each of the main four conditions (10 per gender). The sentence-model pairings were randomized. Prior to each experimental task, participants performed a practice phase that consisted of 16 trials. 

### 2.4. Eye-Tracking and EEG Recordings

A desk-mounted remote SR Research EyeLink 1000 eye-tracker (SR Research, http://sr-research.com) sampling at 1000 Hz was used to monitor eye movements throughout the study. A nine-point automatic calibration was conducted at the beginning of each block, recording each participant’s dominant eye (as determined by the Miles test; [33]). The dominant eye was tracked, but viewing was binocular.

EEG recordings were collected continuously at 512 Hz by an active-two Biosemi system at 72 recording sites: 66 channels in an electrode-cap under the 10/20 system-extended (the default 64 sites plus PO9/PO10 sites), two pairs of electrodes situated on the outer canthi and infra-orbital ridges (to monitor horizontal and vertical eye movements), and one additional pair situated over the mastoids. A common mode sense (CMS) active electrode and a driven right leg (DRL) passive electrode acted as a ground during recording.

### 2.5. Data Processing

Only correct trials with reaction times ±2.5 SD of each participant’s response mean were analyzed. Trials in which participants did not read the contextual sentence were rejected. Specifically, trials with fewer than two fixations within a pre-determined 14.65° × 2.45° sentence region of interest (ROI) were automatically detected, and visually inspected. If participants did not make at least one fixation towards an affectively-valenced word within the sentence, the trial was rejected. This criterion was used because only a small number of sentences were used in this study. Thus, upon continuous repetition participants were able to determine the nature of the sentence by scanning key affective words. Trials in which it was clear that the sentence had been read, but fixations fell outside of the pre-determined sentence ROI due to eye-recording drift, were kept. Furthermore, during the emotional face presentation, participants were required to maintain fixation on the nasion of the face. Any trials in which eye movements were made outside of a 1.37° diameter ROI centered on the nasion were automatically rejected. 

All EEG data was processed offline using EEGLab version 13_6_5b [34] and ERPLab (http://erpinfo.org/erplab) toolboxes in MATLAB version 2014. Recordings were average-referenced offline and synchronized with the eye-tracking recordings. The data were further band-pass filtered (0.01–30 Hz) and epoched into time segments of −100 ms to 800 ms around the onset of the emotional face. Trials with artifacts above or below ±70 µV were automatically detected and removed. Independent component analysis was also conducted for 24 participants in order to remove blink- and eye-movement-contaminated artefacts, and one participant underwent manual cleaning in order to remove remaining artifacts. The average number of trials per condition after pre-processing was 54.1 (SEM = 11.9) and did not vary significantly between the eight conditions (*F*(7,245) = 1.25, *p* = 0.29; Table 1). 

### 2.6. Data Analysis

For each task, the percentage of correct responses was calculated using the trials in which sentences were read and the emotional face was correctly fixated. A response was deemed correct if the correct button press was made and if the Response Time (RT) was more than 150 ms (to avoid anticipatory responses) and less than 2000 ms. Mean response times were calculated for these correct responses, with the additional constraint that RTs longer than 2.5 SD from the overall mean of each participants were rejected. 

Each participant’s average ERP waveforms were individually inspected in order to determine the electrode within each hemisphere for which the N170 was maximal for all conditions (see also [25,35,36,37,38,39]). The N170 was maximal at different electrodes across participants (Table 2), but maximal at the same electrodes across conditions. The N170 peak amplitude was measured bilaterally at the electrodes specified in Table 2 between 120 ms and 200 ms post-stimulus onset. The mean N170 amplitude was also extracted within each hemisphere ±10 ms of the participant’s average peak latency for all conditions within each hemisphere.

For the EPN, mean amplitudes were calculated between 150–250 ms and 250–350 ms at electrodes P9, P10, PO9, and PO10, where it is classically measured (e.g., [23,24,25,26,36,37]). This choice was based on the fact that, while most studies have measured the EPN between 220–350 ms, others typically measure the EPN between 150 and 300 ms (e.g. [26]), and our recent studies suggested that the largest emotion effect was actually found before 200 ms although after the N170 [25,36,37]. We thus decided to measure the EPN at two separate time windows to better capture its time-course. 

Finally, mean amplitudes were computed at two cluster locations, based on inspection of the data and on previously published studies focusing on the LPP: a frontal cluster (AF3, AFz, AF4, F1, Fz, and F2 sites) and a centro-parietal cluster (C1, Cz, C2, CP1, CPz, CP2 and P1, Pz, and P2). Mean amplitudes were extracted at these electrodes between 250–350 ms and 350–450 ms (given the RT pattern, neural activity beyond 450ms would certainly be contaminated by motor preparation artifacts). Because the LPP is classically measured between 400 and 600 ms, we prefer to refer here to a LPP-like component due to its similar scalp distribution but different timing of occurrence. The choice of two time windows was driven both by visual inspection of the data and by prior existing literature in other domains suggesting an early and a late LPP occurring at different timing (e.g., [40,41]). 

Separate repeated measures analyses of variance (ANOVAs) were conducted for the percentage of correct responses, mean RT, as well as for each ERP component individually for each time window. Within-subject factors included (2) task, (2) face emotion, and (2) congruency (with preceding sentence), for all analyses. For N170 and EPN, additional factors were hemisphere (2) and electrodes (2), and for the LPP, an additional factor was clusters (2: frontal, Centro-parietal). Finally, for both EPN and LPP, an additional factor was time (2). All ANOVAs used greenhouse-Geisser adjusted degrees of freedom when the Mauchly’s test of sphericity was significant. Follow-up ANOVAs were conducted when three or four-way interactions were found. Pairwise comparisons were Bonferroni-corrected. 

## 3. Results

### 3.1. Behavioural Results

For the behavioural analyses, data from three participants were lost, resulting in a final sample of *N* = 33. 

#### 3.1.1. Percentage of Correct Responses

**Figure 2:**
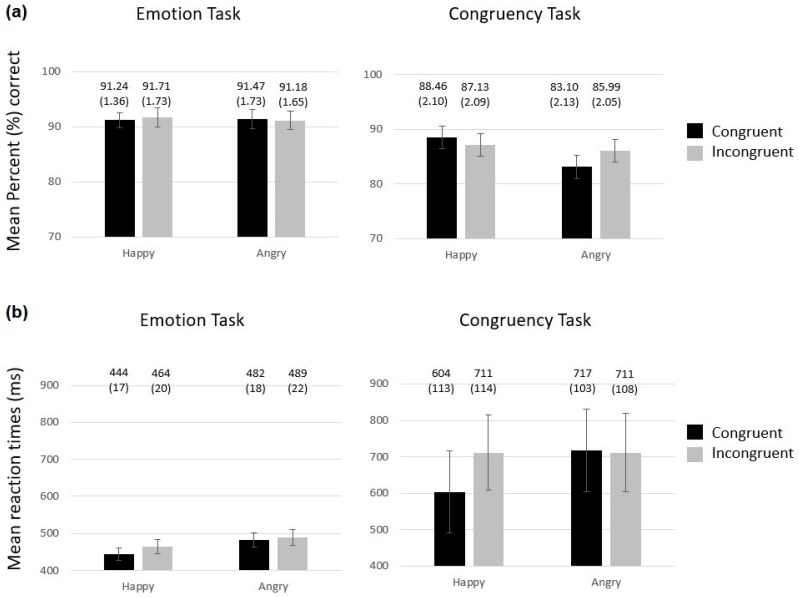
Percentage of correct responses (**a**) and mean response times (**b**) for each face emotion (happy or angry) and congruency of that emotion with the preceding sentence, for each task. Condition means (with standard error (SE) of the mean in parenthesis) are reported above each bar. A small effect of congruency was found for angry faces in the congruency task for correct responses and an effect of congruency was found on Response Times (RTs) for happy faces in the congruency task.

As can be seen on Figure 2A, participants’ performance was overall higher in the emotion than in the congruency task (main effect of task, *F*(1,32) = 11.53, *p* = 0.002, Mean Squared Error *MSE* = 156.3, and *ηp*^2^ = 0.265). Significant effects of emotion (*F*(1,32) = 16.49, *p* < 0.0001, *MSE* = 11.53, and *ηp*^2^ = 0.340), task by emotional interaction (*F*(1,32) = 12.36, *p* = 0.001, *MSE* = 12.85, and *ηp*^2^ = 0.279), emotion by congruency interaction (*F*(1,32) = 4.2, *p* = 0.049, *MSE* = 11.77, and *ηp*^2^ = 0.116), and the three-way interaction of task by emotion by congruency (*F*(1,32) = 6.25, *p* = 0.018, *MSE* = 16.29, and *ηp*^2^ = 0.163), were found. Therefore, the analyses were run for each task separately. No significant effects were found for the emotion task (*p*s > 0.4). However, for the congruency task, the main effects of emotion (*F*(1,32) = 27.32, *p* < 0.0001, *MSE* = 12.75, and *ηp*^2^ = 0.461), and the emotion by congruency interaction (*F*(1,32) = 8.06, *p* = 0.008, *MSE* = 18.18, and *ηp*^2^ = 0.201) were significant. The latter interaction reflected a congruency effect for angry faces (Figure 2A), with slightly better performances for incongruent than congruent trials (*t*(32) = −2.41, *p* = 0.021, Bonferroni corrected *p* = 0.025), while no difference was found for happy faces (*t*(32) = 1.35, *p* = 0.187).

#### 3.1.2. Mean Response Times (ms)

As can be seen on Figure 2B, participants tended to respond slower in the congruency than in the emotion task (main effect of task, *F*(1,32) = 3.91, *p* = 0.057, *MSE* = 786089.1, and *ηp*^2^ = 0.109), but were also a lot more variable in the congruency task, as reflected by the much larger standard errors for this task. Responses were also overall faster for happy than angry faces (main effect of emotion (*F*(1,32) = 41.61, *p* < 0.0001, *MSE* = 13118.6, and *ηp*^2^ = 0.565). Significant main effect of congruency (*F*(1,32) = 31.31, *p* < 0.0001, *MSE* = 2169.9, and *ηp*^2^ = 0.495), task by congruency interaction (*F*(1,32) = 14.01, *p* = 0.001, *MSE* = 1599.1, and *ηp*^2^ = 0.305), emotion by congruency interaction (*F*(1,32) = 73.52, *p* < 0.0001, *MSE* = 894.9, and *ηp*^2^ = 0.697), and the three-way interaction of task by emotion by congruency (*F*(1,32) = 25.6, *p* < 0.0001, *MSE* = 1610.6, and *ηp*^2^ = 0.444), were also found. Therefore, follow-up analyses were run for each task separately. 

For the congruency task, in addition to the significant main effect of emotion (*F*(1,32) = 21.9, *p* < 0.0001, *MSE* = 4890.5, and *ηp*^2^ = 0.406), the congruency effect (*F*(1,32) = 40.65, *p* < 0.0001, *MSE* = 2070.9, and *ηp*^2^ = 0.560) and the emotion by congruency interaction (*F*(1,32) = 51.58, *p* < 0.0001, *MSE* = 2047.6, and *ηp*^2^ = 0.617) were significant. The latter interaction reflected a congruency effect for happy faces only (Figure 2B), with faster responses for congruent than incongruent trials (*t*(32) = −12.03, *p* < 0.0001), while no congruency effect was found for angry faces (*t*(32) = 0.465, *p* = 0.645). For the emotion task, the main effect of emotion was significant (*F*(1,32) = 33.04, *p* < 0.0001, *MSE* = 1004.5, and *ηp*^2^ = 0.508) but the effect of congruency (*F*(1,32) = 3.63, *p* = 0.066, *MSE* = 1697.9, *ηp*^2^ = 0.102) and the emotion by congruency interaction *F*(1,32) = 3.12, *p* = 0.087, *MSE* = 457.9, and *ηp*^2^ = 0.089) were insignificant. 

### 3.2. ERP Results

#### 3.2.1. N170 Component

The N170 could not be clearly identified for one participant, leaving *N* = 35 for this analysis.

For the N170 peak amplitude, only the task by congruency by hemisphere interaction was significant (*F*(1,34) = 5.71, *p* = 0.023, *MSE* = 0.503, and *ηp*^2^ = 0.144). When each task was analyzed separately, the congruency by hemisphere interaction was significant only for the emotion task (*F*(1,34) = 4.65, *p* = 0.038, *MSE* = 0.925, and *ηp*^2^ = 0.12), reflecting a trend for an effect of congruency on the left hemisphere. However, no further post-hoc ANOVA or t-tests were significant. Similarly, when the mean N170 amplitude was used (calculated within ±10 ms around the peak of each participant), only the task by congruency by hemisphere interaction was significant (*F*(1,34) = 5.67, *p* = 0.023, *MSE* = 0.637, and *ηp*^2^ = 0.143) but the analysis of each task separately did not reveal any significant effect. 

#### 3.2.2. EPN Component

The 2 (Time) × 2 (Task) × 2 (Emotion) × 2 (congruency) × 2 (electrodes) × 2 (Hemisphere) omnibus ANOVA revealed interactions between time and emotion (*F*(1,35) = 45.55, *p* < 0.0001, *MSE* = 2.021, and *ηp*^2^ = 0.565) and time and congruency (*F*(1,35) = 29.99, *p* < 0.0001, *MSE* = 0.433, and *ηp*^2^ = 0.462). Therefore, the analyses were re-run for each time window separately.

**Figure 3:**
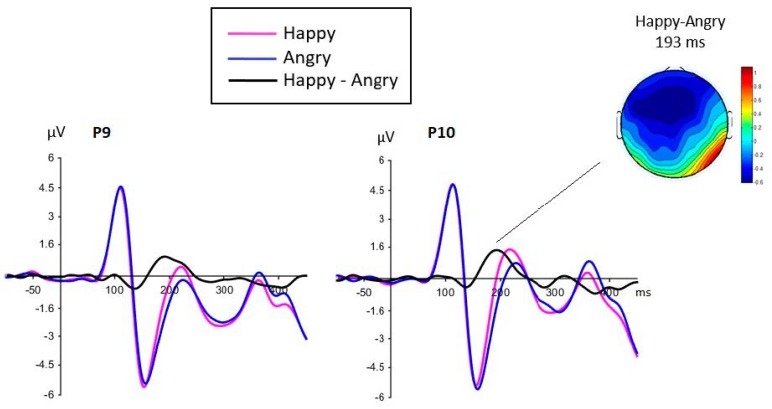
Face emotion effect seen between 150–250 ms at posterior sites, representing the earlier posterior negativity (EPN). The emotion effect was maximum around 193 ms, i.e. after the N170 and around the P2 component (the topographic map represents the voltage at 193 ms for the Happy minus Angry grand average difference). The effect of emotion was not significant for the N170.

Between 150 and 250ms, the analysis of the mean amplitudes at posterior sites revealed a main effect of face emotion (*F*(1,35) = 34.58, *p* < 0.0001, *MSE* = 3.33, and *ηp*^2^ = 0.497), with more negative amplitudes for angry than happy faces, reflecting a classic EPN (Figure 3). A main effect of electrode (*F*(1,35) = 84.12, *p* = 0.0001, *MSE* = 10.23, and *ηp*^2^ = 0.706) was due to more negative amplitudes at P9/10 compared to PO9/PO10 electrodes. We also found weak interactions of congruency by electrode (*F*(1,35) = 5.86, *p* = 0.021, *MSE* = 0.255, and *ηp*^2^ = 0.144), task by emotion by congruency by electrode (*F*(1,35) = 4.86, *p* = 0.034, *MSE* = 0.327, and *ηp*^2^ = 0.122), and task by congruency by electrode by hemisphere (*F*(1,35) = 6.52, *p* = 0.015, *MSE* = 0.225, and *ηp*^2^ = 0.157), but these were most likely spurious effects and follow-up analyses did not reveal any significant and meaningful effects. 

**Figure 4:**
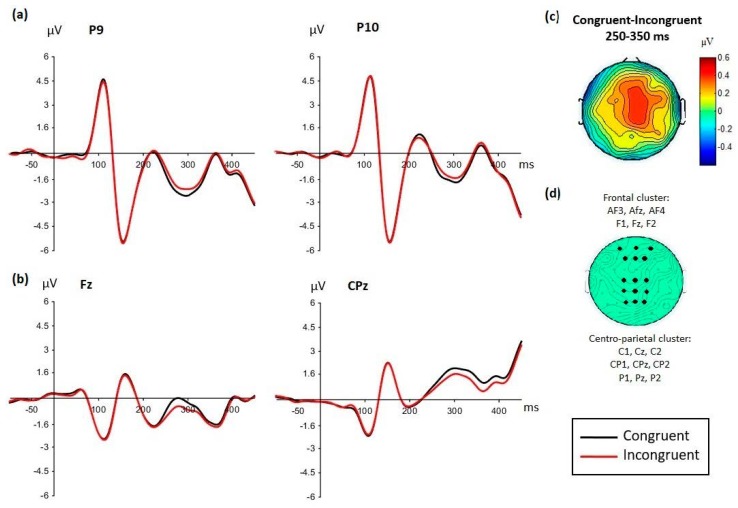
(**a**) Main effect of congruency seen between 250 and 350 ms at posterior sites P9/P10, with more negative amplitudes for congruent than incongruent conditions (group grand average displayed). (**b**) This effect was seen polarity-reversed at frontal and centro-parietal sites (here exemplified by Fz and CPz electrodes), where amplitudes were now more positive for congruent than incongruent conditions. (**c**) The topographic map illustrates the distribution of the congruency effect (congruent minus incongruent), averaged between 250 and 350 ms, and (**d**) the green map shows the two electrode clusters focused on the statistical analyses for the late positive potential (LPP)-like components (frontal and centro-parietal clusters).

In contrast, between 250–350 ms, the main effect of face emotion was no longer significant (*F*(1,35) = 1.93, *p* = 0.173) but the main effect of congruency was significant (*F*(1,35) = 18.74, *p* < 0.001, *MSE* = 1.79, *ηp*^2^ = 0.349), with more negative amplitudes for congruent than incongruent conditions (Figure 4a). This effect of congruency was seen polarity reversed at frontal and centro-parietal sites (Figure 4b) and is captured in the analysis of LPP-like components reported next. Besides the main effect of electrode (*F*(1,35) = 68.02, *p* < 0.0001, *MSE* = 8.29, *ηp*^2^ = 0.66), no other effects or interactions were found.

#### 3.2.3. LPP-Like Components

The 2 (Time) × 2 (task) × 2 (Emotion) × 2 (congruency) × 2 (Clusters) omnibus ANOVA revealed interactions between time and task (*F*(1,35) = 15.18, *p* < 0.001, *MSE* = 1.44, and *ηp*^2^ = 0.303); time and congruency (*F*(1,35) = 9.44, *p* = 0.004, *MSE* = 0.488, and *ηp*^2^ = 0.212); time, task, and congruency (*F*(1,35) = 10.21, *p* = 0.003, *MSE* = 0.252, and *ηp*^2^ = 0.226); time, congruency, and cluster (*F*(1,35) = 8.23, *p* = 0.007, *MSE* = 0.480, and *ηp*^2^ = 0.191); and, finally, time, task, congruency, and cluster (*F*(1,35) = 15.61, *p* < 0.001, *MSE* = 0.350, and *ηp*^2^ = 0.309). Therefore, the analyses were re-run for each time window separately, and the statistical results for these analyses are reported in Table 3. We reported only the important main effects and interactions for clarity. 

**Figure 5:**
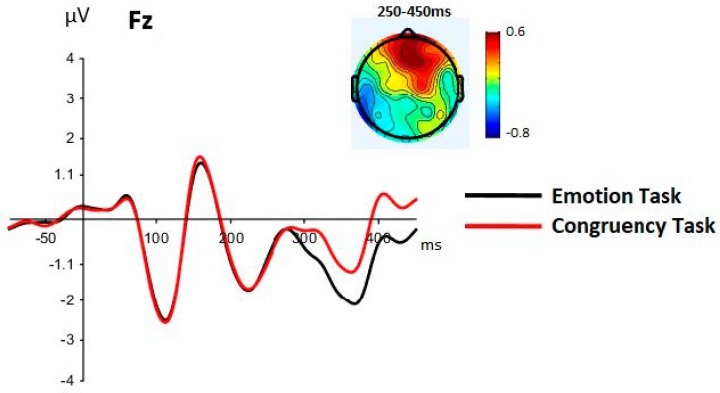
Group grand averaged waveforms for each task (averaged across emotion and congruency). The effect of task was mainly seen at frontal sites (exemplified by Fz here), with larger amplitudes for the congruency task compared to the emotion task, between 250 and 450 ms. The topographic map shows the task effect (congruent minus emotion task) between 250 and 450 ms.

A main effect of task was seen between 350 and 450 ms, but the task by cluster interaction was seen from 250 to 450 ms (Table 3a,d). This interaction was due to the task effect being significant only at frontal sites between 250 and 450 ms (and more strongly between 350 and 450 ms), with less negative amplitudes for the congruency than the emotion task (Figure 5). 

Importantly, the effect of congruency varied with task and emotion but in a seemingly independent way, as no interaction involving the three factors of task, emotion, and congruency was ever found in any analysis. We report in turn the interactions between congruency and task, and the interactions between congruency and emotion. 

**Figure 6:**
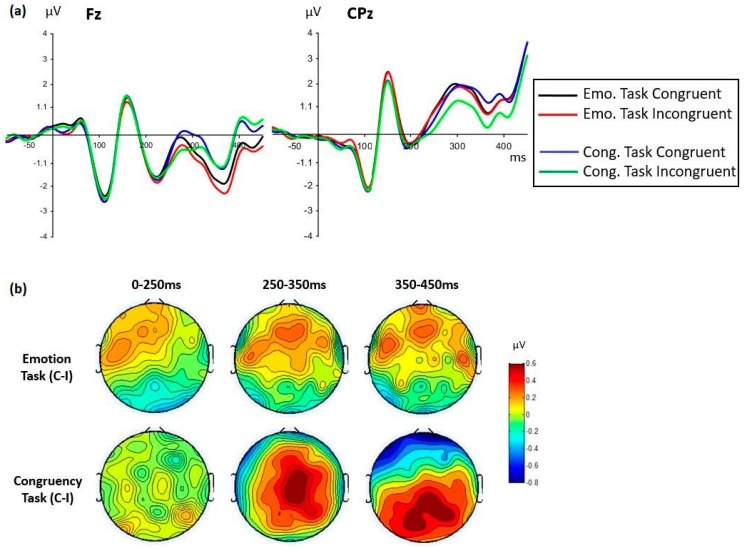
The effect of congruency varied with task. (**a**): group grand averaged waveforms for each task and congruency condition displayed at Fz and CPz sites. (**b**): topographic maps representing the congruency effect (congruent minus incongruent conditions) displayed for each task between face onset (0 ms) and 450 ms. The congruency effect was strongest in the congruency task, with a clear fronto-central then centro-parietal distribution (with polarity reversal at fronto-polar sites), and was clearly weaker and frontally distributed in the emotion task.

The effect of congruency varied depending on the task involved (Table 3c,f,g). Between 250 and 350 ms, the main effect of congruency was due to more positive amplitudes for congruent than incongruent conditions (with reversed-polarity effects at posterior sites, as described above, see Figure 4), and was most pronounced for the congruency task, although statistically significant in both tasks (Table 3e). However, between 350 and 450 ms, this congruency effect interacted with cluster and tasks so analyses were run separately for each cluster (Table 3g). At frontal sites, the task by congruency interaction was due to an opposite effect of congruency depending on the task. In the emotion task, the same effect was seen as previously, with more positive amplitudes for congruent than incongruent conditions, while in the congruency task, the opposite was found, with slightly more positive amplitudes for incongruent compared to congruent conditions (Figure 6). This latter effect was due to the fact that the congruency effect in the congruency task was seen at centro-parietal sites, and was simply in opposite direction at fronto-polar sites (clearly visible on the topographic map in Figure 6 for the 350–450 ms window). At centro-parietal sites, there was no more congruency effect in the emotion task. 

Thus, overall, the congruency effect was seen between 250 and 450 ms and was larger for the congruency than the emotion task (Figure 6 topographic maps). This effect seemed frontally distributed in the emotion task. In the congruency task, the congruency effect was initially seen at frontal, central, and parietal sites and then was more parietally distributed after 350 ms. For both tasks, the effect reflected more positive amplitudes for congruent than incongruent conditions. 

**Figure 7:**
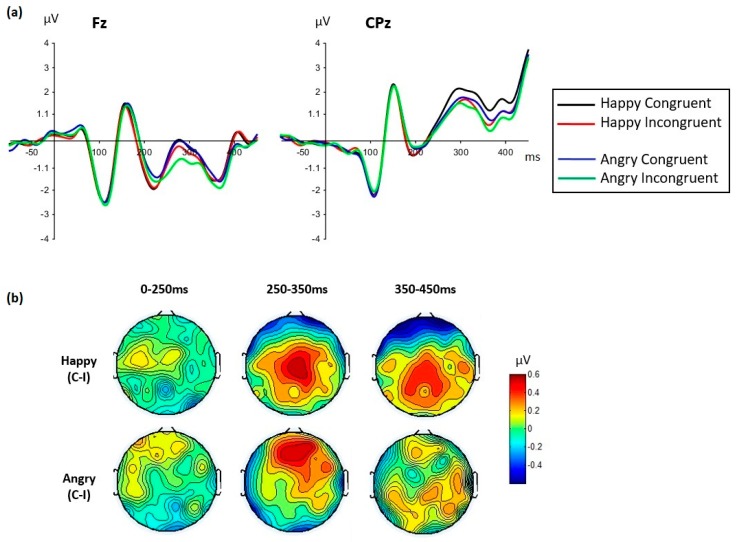
The effect of congruency varied with the face emotion. (**a**)*:* group grand averaged waveforms for each face emotion and congruency condition (averaged across tasks) displayed at Fz and CPz sites. (**b**): topographic maps representing the congruency effect (congruent minus incongruent conditions) are displayed for each face emotion between face onset (0 ms) and 450 ms. The congruency effect displayed a clear centro-parietal distribution for happy face expressions and a clear frontal distribution for angry face expressions, restricted to 250–350 ms.

The congruency effect also varied with the facial expression seen (Figure 7). The emotion by congruency by cluster interaction was significant from 250 to 450 ms (Table 3i), so the analyses were performed for each cluster separately. At frontal sites, a congruency effect was seen for angry expressions. This effect was largest and clearest between 250 and 350 ms, with larger amplitudes for congruent than incongruent conditions (clearly seen on the topographic maps in Figure 7, Table 3i). The congruency effect at frontal sites was insignificant for happy expressions, except weakly during 350–450 ms. At centro-parietal sites, however, the opposite was found, with a clear congruency effect for happy faces from 250 to 450 ms, and no congruency effect for angry faces in any time window. Thus, a different topographical distribution of the congruency effect was seen between happy and angry faces.

## 4. Discussion

This study investigated the effect of congruency between contexts and target facial expressions of emotion under different task demands. The context was situational and presented as a sentence paired with a neutral face, followed by the same face displaying an expression as if reacting to the context. Importantly, eye tracking ensured that every sentence was read in each trial. The two main manipulations of the present within-subject design were the task condition (discriminating the emotion shown by the face or judging its congruency with the context) and the trial condition (congruent vs. incongruent). Behavioral results showed significantly better performance in the emotion task, compared to the congruency task, in terms of response accuracy. Moreover, task comparisons showed an interaction between emotion and congruency only in the congruency task. In this task, a small but significant difference in accuracy was shown in the responses to angry faces that tended to be more accurate in incongruent trials. With respect to reaction times, responses to happy faces were significantly faster in congruent than in incongruent trials for that task. These behavioral results are consistent with our prediction that explicit attention to the congruency between contexts and facial expressions increases its impact on the way the expression is processed and responded to.

Significant effects of congruency and task condition were observed in different ERP components. A main effect of task was clearly manifest at frontal sites between 250 and 450 ms, with more positive amplitudes for the congruency than the emotion task (Figure 5). This difference probably reflects the more elaborate computations required by the congruency task and is consistent with the poorer performance observed in this task in terms of accuracy and response speed. Responding under this condition requires activation of conceptual emotional knowledge that refer to the behaviors that are expected in different emotion-relevant contexts. Among these are the facial expressions that are appropriate or more frequently observed in someone who experiences a specific emotional event. In order to judge the congruency of a target expression, the participant first needs to identify the expressed emotion while she keeps contextual information active in working memory and then integrate both pieces of information. Thus, explicit consideration of how the target expression and the context are related is required. In contrast, the emotion discrimination task only requires taking into account the information provided by the target face and identifying it as expressing joy or anger. These effects of task demands are consistent with the results of other studies that have reported sensitivity of the LPP to cognitive effort and task difficulty (e.g., [42,43]). Of special relevance are studies that have revealed specific modulation of the frontal component of the LPP by effortful emotion regulation strategies [44,45,46]. For example, in the study by Shafir et al. [46], reappraisal compared to distraction strategies was associated with larger frontal LPP amplitudes. This effect was observed in the presence of negative stimuli of high emotional intensity, but not of stimuli of low intensity, that is, in the situation that required superior cognitive effort. Although reappraisal and congruency judgment require different computations, both coincide in requiring cognitive effort and explicit consideration of emotionally relevant information. 

Effects of congruency appeared at latencies and locations corresponding to the EPN and LPP components that have previously been found to be sensitive to emotion and affective congruency. However, the absence of such an effect on earlier perceptual components suggests that in the present study early visual processing of target faces was not modulated by the immediately preceding context. Only a trend for a congruency effect restricted to the emotion task and localized in the left hemisphere was found on the N170, a component associated with the early stages of face processing. This is in contrast to previous studies that have revealed congruency effects on this component [3,5,7]. Given the similarities between the procedure employed in the present study and in those by Dieguez-Risco et al. [6,7], one would have expected to find similar results. However, there were also some methodological differences that might have contributed to this discrepancy. One factor of potential importance is the strict control of eye gaze in the present experiment by means of an eye-tracker, which was implemented given the growing literature suggesting modulation of the N170 component with fixation location [35,36,37,38,39,47]. This procedure ensured that differences in gaze position would not modulate the processing of the target faces. Under such circumstances, it appears that early perceptual processes as indexed by the N170 are not influenced by the preceding context, a result that will need to be replicated. 

The overall effect of congruency was observed with latency and localization corresponding to the EPN component (250–350 ms at posterior sites), but was seen polarity-reversed at frontal and centro-parietal sites under the form of an LPP-like component (Figure 4). At those sites, larger amplitudes were seen for congruent than incongruent conditions. Importantly, this effect of congruency between contexts and target faces was independently modulated by the task condition and by the emotional expression. 

First, the magnitude and spatial distribution of the congruency effect varied depending on task demands. As predicted, the largest effect of congruency was observed in the congruency task, with a predominantly centro-parietal distribution that was seen polarity-reversed at fronto-polar sites (clearly seen on Figure 6 topographic maps between 350 and 450 ms). In contrast, the congruency effect in the emotion discrimination task was only frontally distributed and much weaker. This difference is consistent with the behavioral results previously discussed, which revealed significant congruency effects only in the congruency task. This finding is consistent with our hypothesis that congruency should have a larger effect precisely in the congruency task that requires explicit consideration of the relation between the expression shown by the target face and the situational context in which it is perceived. The demands of that task lead to the operation of top-down processes that involve deliberate access and use of conceptual knowledge referred to by specific emotions and the reactions usually associated with them. The fact that congruency effects were also obtained in the emotion task (albeit to a smaller degree) means that situational contexts influence processing of facial expressions, even when contextual information is not relevant for the task at hand. However, the different topographical distributions of these effects suggest different underlying generators and time courses for the congruency effect, and thus the operation of different underlying mechanisms, depending on the task demands. Overall, the LPP-like results revealed a dynamic, complex picture that suggests different processing operations driven by the different demands imposed by the emotion discrimination and congruency tasks. In other words, processing of the target expression was modulated by the context in different ways depending on the specific task at hand.

The timing and spatial distribution of the congruency effects were also different for angry and happy target faces. A congruency effect was seen for angry faces at frontal sites during the LPP-like component. However, the corresponding congruency effect showed a centro-parietal distribution in the case of happy faces. If the frontal LPP-like component is an index of effortful processing operations, then it might be concluded that processing the contextual congruency of an emotional expression is a more demanding task for angry than for happy faces. The different distribution and timing of these congruency effects again suggest different underlying generators depending on the face expression seen. 

The suggestion that context congruency judgments require more cognitive effort in the case of angry, compared to happy, faces is also consistent with the behavioral results that showed a significant effect for angry faces in the congruency task. More specifically, judgment accuracy was higher in angry faces incongruent trials. Similar results have been reported before [7,9], showing more accurate and/or faster responses to angry faces in happiness than in anger contexts. This result, which might seem counterintuitive at first sight, can be understood in terms of the different specificities of the facial expressions of positive and negative emotions. According to this view, judging if an angry expression is contextually appropriate would be an especially difficult task because it involves discriminating between a variety of negative emotions and their corresponding situational and expressive characteristics. In contrast, the fact that a smiling face is appropriate in different happy situations would make the corresponding congruency judgment an easier task. Based on this rationale, a double-check hypothesis has been proposed according to which processing facial expressions of emotion involves a sequential check of valence and emotion category [7,9,14]. According to this hypothesis, a valence check would suffice to judge the contextual congruency of a smiling face (a smiling face is incongruent in any negative context but can be congruent in many positive contexts). However, judging the congruency of an angry face would further require an emotion category check (although affectively negative, an angry face can be incongruent with sadness or fear contexts, for example). 

Of secondary importance, the results corresponding to the EPN component showed the significant effect of emotion, with larger negative amplitudes for angry than happy face targets. This effect replicates the results of previous studies that have reported specific sensitivity of this component to faces showing angry compared to happy expressions [24,26]. Interestingly, this emotion effect was found only between 150 and 250 ms, with a peak seen before 200 ms (Figure 3), a result consistent with recent studies investigating fearful expressions [25,36,37].

A few limitations to this study must be acknowledged. A fairly small number of sentences and individual faces were used and repeated numerous times across the course of the study, which might have elicited fatigue effects in participants and potentially diminished the contextual effects recorded. The gaze-contingent procedure also introduces variability in duration between the end of the sentence reading and the onset of the facial expression, which, in addition to individual variability in reading speed and sentence length and variability, might also influence the contextual effects. These factors, along with the intensity of the emotional expressions seen, might modulate the contextual effects and should be investigated in future studies. 

## 5. Conclusions

The results reported in the present study confirm the modulatory role of situational contexts on the processing of target facial expressions of emotion. These effects were especially prominent in the LPP-like component that is specifically modulated by emotional content and has been previously found to be sensitive to affective congruency [7,17,18,19]. Moreover, our results provide new evidence that this effect of congruency is significantly modulated by task demands. More specifically, larger effects of context-target congruency were observed in an explicit task that required the participants to judge the congruency of a target expression with an immediately preceding context. Smaller congruency effects were also observed in an emotion discrimination task. The different properties of the congruency effects obtained in each of these tasks suggest that they are mediated by different mechanisms and depend on the operation of different neural systems. We propose that while congruency acts via implicit, automatic mechanisms in the case of the emotion task, those observed in the congruency task are based on top-down, deliberate processes that rely on conceptual knowledge about the reactions that are expected from a person that goes through different emotional experiences.

## Figures and Tables

**Table 1:** Mean number of trial per each of the eight conditions (standard deviations in parenthesis).

Task	Conditions	Mean Trial Number (SD)	Min/Max Trial Number
Emotion Task	Happy Congruent	54.06 (11.68)	30/75
	Happy Incongruent	54.08 (12.00)	32/73
	Angry Congruent	54.58 (12.57)	31/75
	Angry Incongruent	54.11 (11.58)	27/76
Congruency Task	Happy Congruent	56.36 (12.17)	36/76
	Happy Incongruent	54.58 (12.02)	27/74
	Angry Congruent	52.39 (11.5)	23/71
	Angry Incongruent	53.03 (12.31)	29/71

**Table 2:** Participant distribution for maximal N170 peak electrodes (all conditions) in the left and right hemispheres.

Left Hemisphere		Right Hemisphere	
Electrode	Number of Participants	Electrode	Number of Participants
P7	1		
PO7	1	TP10	1
PO9	4	PO10	6
P9	30	P10	29

**Table 3:** Statistical results obtained for the analyses on mean amplitudes calculated over two time windows at two clusters (frontal and centro-parietal clusters).

Statistical Effects	250–350 ms	350–450 ms
**(a) Task**	*F*(1,35) = 0.831, *MSE* = 3.21, *p* = 0.36, *ηp*^2^ = 0.023	*F*(1,35) = 12.32, *MSE* = 5.53, *p* = 0.001, *ηp*^2^ = 0.260
**(b) Emotion**	*F*(1,35) = 3.04, *MSE* = 1.56, *p* = 0.09, *ηp*^2^ = 0.080	*F*(1,35) = 7.43, *MSE* = 2.01, *p* = 0.010, *ηp*^2^ = 0.175
**(c) Congruency**	*F* (1,35) = 43.27, *MSE* = 0.75, *p* < 0.0001, *ηp*^2^ = 0.553 Congruent > incongruent	*F*(1,35) = 6.07, *MSE* = 1.16, *p* = 0.019, *ηp*^2^ = 0.148 Congruent > incongruent
**(d) Task × cluster**	*F*(1,35) = 4.57, *MSE* = 5.05, *p* = 0.04, *ηp*^2^ = 0.115	*F*(1,35) = 8.55, *MSE* = 5.21, *p* = 0.006, *ηp*^2^ = 0.196
*Frontal cluster* Task effect	*F*(1,35) = 5.29, *MSE* = 3.92, *p* = 0.028, *ηp*^2^ = 0.131 Congruency task > emotion task	*F*(1,35) = 16.74, *MSE* = 6.65, *p* = 0.001, *ηp*^2^ = 0.324 Congruency task > emotion task
*Centro-parietal cluster* Task effect	*F*(1,35) = 1.15, *MSE* = 4.35, *p* = 0.29, *ηp*^2^ = 0.032	*F*(1,35) = 0.31, *MSE* = 4.08, *p* = 0.58, *ηp*^2^ = 0.009
**(e) Task × congruency**	*F*(1,35) = 5.49, *MSE* = 0.945, *p* = 0.025, *ηp*^2^ = 0.136	*F*(1,35) = 0.00, *MSE* = 0.981, *p* = 0.991, *ηp*^2^ = 0.000
*Emotion task* Congruency effect	*F*(1,35) = 9.22, *MSE* = 0.634, *p* = 0.005, *ηp*^2^ = 0.208 Congruent > incongruent	
*Congruency task* Congruency effect	*F*(1,35) = 29.97, *MSE* = 1.06, *p* < 0.0001, *ηp*^2^ = 0.461 Congruent > incongruent	
**(f) Congruency × cluster**	*F*(1,35) = 0.113, *MSE* = 0.942, *p* = 0.739, *ηp*^2^ = 0.003	*F*(1,35) = 8.33, *MSE* = 1.18, *p* = 0.007, *ηp*^2^ = 0.192
**(g) Task × congruency × cluster**	*F*(1,35) = 2.63, *MSE* = 1.41, *p* = 0.070, *ηp*^2^ = 0.113	*F* (1,35) = 12.86, *MSE* = 2.13, *p* = 0.001, *ηp*^2^ = 0.269
*Frontal cluster* Task × congruency		*F*(1,35) = 8.01, *MSE* = 1.70, *p* = 0.008, *ηp*^2^ = 0.186
		*Emo. task (congruency effect):* *F*(1,35) = 4.75, *MSE* = 1.18, *p* = 0.036, *ηp*^2^ = 0.119 Congruent > incongruent
		*Cong. task (congruency effect):* *F*(1,35) = 4.59, *MSE* = 1.77, *p* = 0.039, *ηp*^2^ = 0.116 Incongruent > congruent
*Centro-parietal cluster* Task × congruency		*F*(1,35) = 9.77, *MSE* = 1.41, *p* = 0.004, *ηp*^2^ = 0.218
		*Emo. Task: no effect of congruency* (*p* = 0.78)
		*Cong. task (congruency effect):* *F*(1,35) = 20.42, *MSE* = 1.49, *p* < 0.0001, *ηp*^2^ = 0.368 Congruent > incongruent
**(h) Emotion × congruency**	*F*(1,35) = 4.41, *MSE* = 0.361, *p* = 0.043, *ηp*^2^ = 0.112	*F*(1,35) = 0.60, *MSE* = 0.750, *p* = 0.444, *ηp*^2^ = 0.017
**(i) Emotion × congruency × cluster**	*F* (1,35) = 15.43, *MSE* = 1.17, *p* = 0.001, *ηp*^2^ = 0.306	*F* (1,35) = 6.88, *MSE* = 1.55, *p* = 0.013, *ηp*^2^ = 0.164
*Frontal cluster* Emotion × congruency	*F* (1,35) = 13.78, *MSE* = 1.10, *p* = 0.001, *ηp*^2^ = 0.283	*F* (1,35) = 4.65, *MSE* = 1.66, *p* = 0.038, *ηp*^2^ = 0.117
	*Happy faces (no congruency effect):* *F*(1,35) = 0.008, *MSE* = 0.677, *p* = 0.92, *ηp*^2^ < 0.001	*Happy faces (congruency effect):* *F*(1,35) = 4.29, *MSE* = 1.13, *p* = 0.046, *ηp*^2^ = 0.109 Congruent > incongruent
	*Angry faces (congruency effect):* *F*(1,35) = 28.65, *MSE* = 1.03, *p* < 0.0001, *ηp*^2^ = 0.450 Congruent > incongruent	*Angry faces (no congruency effect):* *F*(1,35) = 1.68, *MSE* = 1.77, *p* = 0.203, *ηp*^2^ = 0.046
*Centro-parietal cluster* Emotion x congruency	*F*(1,35) = 10.42, *MSE* = 0.431, *p* = 0.003, *ηp*^2^ = 0.229	*F*(1,35) = 5.29, *MSE* = 0.637, *p* = 0.027, *ηp*^2^ = 0.131
	*Happy faces (congruency effect):* *F*(1,35) = 31.88, *MSE* = 0.638, *p* < 0.0001, *ηp*^2^ = 0.477 Congruent > incongruent	*Happy faces (congruency effect):* *F*(1,35) = 38.14, *MSE* = 0.462, *p* < 0.0001, *ηp*^2^ = 0.522 Congruent > incongruent
	*Angry faces (no congruency effect):* *F*(1,35) = 2.61, *MSE* = 0.878, *p* = 0.11, *ηp*^2^ = 0.069	*Angry faces (no congruency effect):* *F*(1,35) = 2.01, *MSE* = 1.27, *p* = 0.16, *ηp*^2^ = 0.055

## Appendix A. Sentences Used as Context Primes

**Negative sentences (anger inducing)**
1. He/she has been lining up for hours when someone cuts in before him/her
2. He/she has just realized that his/her new mobile phone was stolen
3. He/she bought an appliance that is broken and they will not refund the money
4. He/she has just noticed someone has vandalized his/her brand new car
5. He/she is denied concert entrance because he/she was sold fake tickets
6. His/her friend borrowed a hundred dollars and never paid him/her back
7. He/she catches his/her partner cheating on him/her with his/her best friend
8. He/she has missed his/her vacation trip because of an airport strike
9. They have been speaking badly of his/her mother to infuriate him/her
10. He/she is at a bus stop and the bus driver refuses to stop
**Positive sentences (Joy inducing)**
1. He/she has won two tickets to see his/her favorite band in concert
2. He/she has received the great promotion that he/she wanted at work
3. He/she is finally given the keys to his/her very first house
4. He/she is enjoying the first day of his/her much-needed holidays
5. He/she has been selected amongst many for a very important casting
6. He/she finally got a date with the person of his/her dreams
7. His/her country’s soccer team has just won the world cup final
8. He/she has just received a very important scholarship to study abroad
9. He/she has just won the Outstanding-Graduate-of-the-Year Award
10. He/she finds out his/her child received the best grades of his class

## References

[1] Barrett L.F., Mesquita B., Gendron M. (2011). Context in emotion perception. Curr. Dir. Psychol. Sci..

[2] Wieser M.J., Brosch T. (2012). Faces in Context: A Review and Systematization of Contextual Influences on Affective Face Processing. Front. Psychol..

[3] Hietanen J.K., Astikainen P. (2013). N170 response to facial expressions is modulated by the affective congruency between the emotional expression and preceding affective picture. Biol. Psychol..

[4] Righart R., De Gelder B. (2008). Rapid influence of emotional scenes on encoding of facial expressions: An ERP study. Soc. Cogn. Affect. Neurosci..

[5] Righart R., De Gelder B. (2008). Recognition of facial expressions is influenced by emotional scene gist. Cogn. Affect. Behav. Neurosci..

[6] Diéguez-Risco T., Aguado L., Albert J., Hinojosa J.A. (2013). Faces in context: Modulation of expression processing by situational information. Soc. Neurosci..

[7] Diéguez-Risco T., Aguado L., Albert J., Hinojosa J.A. (2015). Judging emotional congruency: Explicit attention to situational context modulates processing of facial expressions of emotion. Biol. Psychol..

[8] Kim H., Somerville L.H., Johnstone T., Polis S., Alexander A.L., Shin L.M., Whalen P.J. (2004). Contextual Modulation of Amygdala Responsivity to Surprised Faces. J. Cogn. Neurosci..

[9] Aguado L., Martínez-García N., Solís-Olce A., Diéguez-Risco T., Hinojosa J.A. (2018). Effects of affective and emotional congruency on facial expression processing under different task demands. Acta Psychol..

[10] Bentin S., Allison T., Puce A., Perez E., McCarthy G. (1996). Electrophysiological Studies of Face Perception in Humans. J. Cogn. Neurosci..

[11] Eimer M. (2011). The Face-Sensitivity of the N170 Component. Front. Hum. Neurosci..

[12] Itier R.J., Taylor M.J. (2004). N170 or N1? Spatiotemporal Differences between Object and Face Processing Using ERPs. Cereb. Cortex.

[13] Rossion B., Jacques C., Luck S.J., Kappenman E.S. (2012). The N170: Understanding the time course of face perception in the human brain. The Oxford Handbook of Event-Related Potential Components.

[14] Aguado L., Dieguez-Risco T., Méndez-Bértolo C., Pozo M.A., Hinojosa J.A. (2013). Priming effects on the N400 in the affective priming paradigm with facial expressions of emotion. Cogn. Affect. Behav. Neurosci..

[15] Hinojosa J., Mercado F., Carretié L. (2015). N170 sensitivity to facial expression: A meta-analysis. Neurosci. Biobehav. Rev..

[16] Cuthbert B.N., Schupp H.T., Bradley M.M., Birbaumer N., Lang P.J. (2000). Brain potentials in affective picture processing: Covariation with autonomic arousal and affective report. Boil. Psychol..

[17] Schupp H.T., Öhman A., Junghöfer M., Weike A.I., Stockburger J., Hamm A.O. (2004). The Facilitated Processing of Threatening Faces: An ERP Analysis. Emotion.

[18] Herring D.R., Taylor J.H., White K.R., Crites Jr S.L. (2011). Electrophysiological responses to evaluative priming: The LPP is sensitive to incongruity. Emotion.

[19] Hinojosa J.A., Carretié L., Méndez-Bértolo C., Míguez A., Pozo M.A. (2009). Arousal modulates affective priming: An event-related potentials study. NeuroImage.

[20] Werheid K., Alpay G., Jentzsch I., Sommer W. (2005). Priming emotional facial expressions as evidenced by event-related brain potentials. Int. J. Psychophysiol..

[21] Bradley M.M., Hamby S., Löw A., Lang P.J. (2007). Brain potentials in perception: Picture complexity and emotional arousal. Psychophysiology.

[22] Herbert C., Junghöfer M., Kissler J. (2008). Event related potentials to emotional adjectives during reading. Psychophysiology.

[23] Schupp H.T., Markus J., Weike A.I., Hamm A.O. (2003). Emotional Facilitation of Sensory Processing in the Visual Cortex. Psychol. Sci..

[24] Schupp H.T., Junghöfer M., Weike A.I., Hamm A.O. (2004). The selective processing of briefly presented affective pictures: An ERP analysis. Psychophysiology.

[25] Itier R.J., Neath-Tavares K.N. (2017). Effects of task demands on the early neural processing of fearful and happy facial expressions. Brain Res..

[26] Rellecke J., Sommer W., Schacht A. (2012). Does processing of emotional facial expressions depend on intention? Time-resolved evidence from event-related brain potentials. Boil. Psychol..

[27] Klauer K.C., Musch J., Mush J., Klauer K.C. (2003). Affective priming: Findings and theories. The psychology of Evaluation: Affective Processes in Cognition and Emotion.

[28] De Houwer J., Hermans D., Rothermund K., Wentura D. (2002). Affective priming of semantic categorisation responses. Cogn. Emot..

[29] Klauer K.C., Musch J. (2001). Does sunshine prime loyal? Affective priming in the naming task. Q. J. Exp. Psychol. Sect. A.

[30] Klinger M.R., Burton P.C., Pitts G.S. (2000). Mechanisms of unconscious priming: I. Response competition, not spreading activation. J. Exp. Psychol. Learn. Mem. Cogn..

[31] Barrett L.F., Kensinger E.A. (2010). Context Is Routinely Encoded During Emotion Perception. Psychol. Sci..

[32] Tottenham N., Tanaka J.W., Leon A.C., McCarry T., Nurse M., Hare T.A., Marcus D.J., Westerlund A., Casey B., Nelson C. (2009). The NimStim set of facial expressions: Judgments from untrained research participants. Psychiatry Res..

[33] Miles W.R. (1930). Ocular dominance in human adults. J. Gen. Psychol..

[34] Delorme A., Makeig S. (2004). EEGLAB: An open source toolbox for analysis of single-trial EEG dynamics including independent component analysis. J. Neurosci. Methods.

[35] Itier R.J., Preston F. (2018). Increased Early Sensitivity to Eyes in Mouthless Faces: In Support of the LIFTED Model of Early Face Processing. Brain Topogr..

[36] Neath K.N., Itier R.J. (2015). Fixation to features and neural processing of facial expressions in a gender discrimination task. Brain Cogn..

[37] Neath-Tavares K.N., Itier R.J. (2016). Neural processing of fearful and happy facial expressions during emotion-relevant and emotion-irrelevant tasks: A fixation-to-feature approach. Boil. Psychol..

[38] Nemrodov D., Anderson T., Preston F.F., Itier R.J. (2014). Early sensitivity for eyes within faces: A new neuronal account of holistic and featural processing. NeuroImage.

[39] Parkington K.B., Itier R.J. (2018). One versus two eyes makes a difference! Early face perception is modulated by featural fixation and feature context. Cortex.

[40] Naumann E., Bartussek D., Diedrich O., Laufer M.E. (1992). Assessing cognitive and affective information processing functions of the brain by means of the late positive complex of the event-related potential. J. Psychophysiol..

[41] Ruchkin D.S., Johnson R., Mahaffey D., Sutton S. (1988). Toward a Functional Categorization of Slow Waves. Psychophysiology.

[42] Kopf J., Dresler T., Reicherts P., Herrmann M.J., Reif A. (2013). The Effect of Emotional Content on Brain Activation and the Late Positive Potential in a Word n-back Task. PLoS ONE.

[43] Matsuda I., Nittono H. (2015). Motivational significance and cognitive effort elicit different late positive potentials. Clin. Neurophysiol..

[44] Bernat E.M., Cadwallader M., Seo D., Vizueta N., Patrick C.J. (2011). Effects of Instructed Emotion Regulation on Valence, Arousal, and Attentional Measures of Affective Processing. Dev. Neuropsychol..

[45] Moser J.S., Hartwig R., Moran T.P., Jendrusina A.A., Kross E. (2014). Neural markers of positive reappraisal and their associations with trait reappraisal and worry. J. Psychol..

[46] Shafir R., Schwartz N., Blechert J., Sheppes G. (2015). Emotional intensity influences pre-implementation and implementation of distraction and reappraisal. Soc. Cogn. Affect. Neurosci..

[47] De Lissa P., McArthur G., Hawelka S., Palermo R., Mahajan Y., Hutzler F. (2014). Fixation location on upright and inverted faces modulates the N170. Neuropsychologia.

